# Why Do Some Depressive Patients Have Suicidal Ideation but Others Not? Suicidal Ideation From the Perspective of Affective Neuroscience Personality Traits

**DOI:** 10.1002/brb3.70077

**Published:** 2024-10-08

**Authors:** Yasemin Hoşgören Alici, Selvi Ceran, Jamal Hasanli, Gonca Asut, Beren Özel, Zehra Ucar Hasanli, Gökçe Saygi, Arda Bağcaz, Emre Misir

**Affiliations:** ^1^ Department of Psychiatry Baskent University Faculty of Medicine Ankara Turkey; ^2^ Department of Psychiatry Ankara University Faculty of Medicine Ankara Turkey; ^3^ Department of Otorhinolaryngology University of Health Sciences Etlik City Hospital Ankara Turkey

**Keywords:** affective neuroscience, hopelessness, major depressive disorder, suicide

## Abstract

**Introduction:**

Although major depression is the disorder most frequently associated with suicidal behavior, it is unclear that major depressive disorder patients may develop suicidal ideation. Basic affective system theory may provide a novel and beneficial viewpoint in this field. The goal of this study was to investigate the basic affective system in relation to suicidal ideation in individuals with depression.

**Method:**

The study population comprised 160 participants who had been formally diagnosed with major depressive disorder. Participants were divided into two groups according to whether they had suicide ideation (depression with suicide [DS]) (*N* = 93) or not (depression with no suicidal ideation [DNS]) (*N* = 67). The Beck Depression Inventory (BDI), the Suicide Probability Scale (SPS), the Beck Hopelessness Scale (BHS), and the Affective Neuroscience Personality Scale (ANPS) were applied. Statistical Product and Service Solutions (SPSS) 24 and the SPSS macroprocess, which were specifically developed for assessing complex models including serial mediators, were used to analyze the data.

**Results:**

The mean age of all participants was 31.1 ± 11 years, and most of them were female (65%). The DS group had a higher family history of psychiatric medication use and suicidal attempts. In addition, BDI, BHS, and SPS total scores were higher, as well as ANGER subscale scores were higher in the DS group. In mediation analysis, the ANGER subscale significantly predicted the presence of suicide ideation. We observed a direct effect of the ANGER subscale score on suicide ideation as well as an indirect effect of the ANGER subscale score on suicidal ideation via depression severity.

**Conclusion:**

Higher scores on ANGER are associated with suicidal ideation. Neurobiological correlates, including the ANGER system, may be promising in understanding suicidal behaviors.

## Graphical abstract

ANGER basic affect system scores, measured by the affective neuroscience personality scale, are associated with suicidal ideation in major depression patients. The severity of depression also strengthens the relationship between ANGER and suicide ideation.

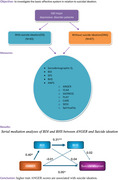

## 1 Introduction

Suicide is a catastrophic behavior for the person, family, and society, causing serious financial and moral harm (Brezo et al. 2006). As a result, risk variables have been thoroughly studied. Suicidal tendencies have been observed to develop over time in the context of a psychiatric disorder. The mental disorder most often related to suicidal ideations is major depressive disorder (MDD), a relatively frequent condition in the general population (Miret et al. 2013). Thus, one of the most critical aspects of managing a patient with depression is recognizing the risk of suicide and the factors that trigger suicide.

The term suicidal behaviors is usually used for both suicide ideation and attempt of suicide, including both uncompleted and completed suicides (Klonsky et al. 2016). Suicidal behaviors emerge from the interplay of trait‐like vulnerability, environmental stressors, and individual triggers involving maladaptive cognitions, behaviors, and affect (Esposito et al. 2003; Mann et al. 1999; Shneidman 1993). Although the etiology of suicide attempts and suicidal ideation may differ, suicidal ideation is one of the most important risk factors for the development of suicide attempts (Klonsky et al. 2016; Nock et al. 2018). Personality traits that significantly influence the development of mental disorders are one of the characteristics that appear to contribute to the development of suicidality. In complex behavioral phenomena like suicidality, personality traits are assumed to play a regulatory role. It is thought that personality traits contribute to the maturation of suicidal ideation, as they represent the person's perception of herself/himself and the world (Baumeister 1990; Dean, Range, and Goggin 1996; Harkness and Lilienfeld 1997). Because this maturing process takes time, there is a chance to intervene in suicidal ideation as a public health concern.

Hopelessness is another factor that plays an important role in suicidality, particularly suicidal ideation (Beck et al. 1985; Baryshnikov et al. 2020; Smith, Alloy, and Abramson 2006). In a meta‐analysis of longitudinal studies, it was found that hopelessness significantly predicted suicidal thoughts and attempts in patients with depression (Ribeiro et al. 2018). In a recent study, although hopelessness was found to be a risk for suicide, it was positively correlated with help‐seeking behavior (Peros and Ward‐Ciesielski 2021). In the presence of hopelessness, an individual's coping style and use of resources for solutions may be affected by personality structure. Therefore, it may be important to consider personality traits when interpreting the relationship between hopelessness and suicide.

Because depression is an affective disorder, it is reasonable to expect that the regulatory function of personality traits on the affect is compromised (Brezo et al. 2006). Understanding the psychobiological underpinnings of personality traits may thus provide useful knowledge to comprehend the psychobiological bases of suicidal ideation. The Affective Neuroscience Personality Scale (ANPS), which is based on affective neuroscience's core affective system theory with reference to the emotional aspect of personality, seems promising for investigating this relationship. *The basic affective system* is a theory developed by Panksepp through brain stimulation and pharmacological experiments. According to this approach, emotions are natural entities that have been preserved in the evolutionary process and embedded in the mammalian system (Ekman, Sorenson, and Friesen 1969; Panksepp 1982). Panksepp claims that there are seven primary affective systems (CARE, PLAY, SEEKING, FEAR, ANGER, SADNESS, and LUST) that regulate mammalian behavior from the bottom up, located in phylogenetically ancient subcortical areas of the mammalian brain (Panksepp 2000). Emotions emerge from subcortical systems, and individual differences are created at cognitive levels after the interaction of these subcortical systems with the environment (Hauser and Akre 2001). Although few emotional systems have been proposed, they provide a rich phenomenology in the process of social learning (Arias et al. 2020). Given that individual differences in emotional dispositions are part of the personality, a personality profile of the primary affective systems allows for the study of the most evolutionarily ancient aspects of personality. The circuits that comprise the basic affective systems and are represented in the personality profile have been largely mapped in terms of underlying neuroanatomical structures and neurotransmitter systems. Currently, the clinical studies utilizing the ANPS mostly focused on depression, bipolar disorder, dysthymia, borderline personality disorders, attention deficit and hyperactivity disorder, suicide, childhood trauma, and social trauma (Carré et al. 2015; Fuchshuber et al. 2019; Malcolm‐Smith et al. 2013; Montag, Elhai, and Davis 2021; Panksepp 2012; Savitz, van der Merwe, and Ramesar 2008; Wernicke et al. 2019; Yovell et al. 2016). Panksepp and Yovell (2014) pointed out that SADNESS, SEEKING, and PLAY circuits are important in understanding depression. In a study conducted with depressive patients, Montag et al. (2017) found that increased FEAR and SADNESS were linked with depressive symptoms. They hypothesized that increased SEEKING activity combined with higher SADNESS and FEAR may cause suicide. Yovell et al. (2016) showed that suicidal ideation regressed after administering ultra‐low dose buprenorphine to serious suicidal patients with no history of substance abuse. They claimed that this may be because ultra‐low dose buprenorphine treatment reduces the hyperactivation of the endorphinergic SADNESS system without reversing the partial shutdown of the aminergic SEEKING system (Panksepp and Yovell 2014).

Revealing the potential relationship between suicide ideation and affective personality dimensions could enable us to explore the neural basis of suicide ideation through a neurobiological perspective that facilitates the examination of personality dimensions using objective methods (such as animal studies). The goal of this study was to investigate the basic affective system in relation to suicidal ideation in individuals with depression. In‐line with this main objective, our hypothesis is that high levels of SADNESS, ANGER, FEAR, and SEEKING with hopelessness are associated with suicidal ideation.

## 2 Materials and Methods

### 2.1 Participants

The sample of the study consisted of 160 patients diagnosed with MDD. The participants who consecutively applied to Başkent University Psychiatry Outpatient Clinic between January, 2022 and January, 2023 were recruited in the study. Patients included in the study underwent a detailed psychiatric interview by an experienced psychiatrist (YHA, BÖ, GA, JH) according to DSM‐5 criteria. The inclusion criteria were being between 18 and 65 years of age, meeting the diagnostic criteria for MDD according to DSM‐5, and having a Beck Depression Inventory (BDI) score > 16. Exclusion criteria were a history of having alcohol or substance use disorder, any psychotic disorder, bipolar disorder, and dementia. Participants were divided into two groups on the basis of whether they had suicidal ideation or not. They were asked whether they had suicidal thoughts currently or at any time in the current episode and grouped accordingly. The study protocol was approved by the Research Ethics Committee of Başkent University (KA22/458). All participants had provided informed consent before inclusion in the study.

### 2.2 Clinical Assessments

#### 2.2.1 Depression Severity

Participants were given the BDI, a 21‐item self‐report questionnaire with each item scored from 1 to 4, to evaluate the severity of depressive symptoms (Beck et al. 1974). It is interpreted as minimal for scores between 0 and 9, mild for scores between 10 and 16, moderate for scores between 17 and 29, and severe for scores between 30 and 63. The scale was translated into Turkish, and validity and reliability studies were conducted (Hisli 1989). The internal consistency for the nonclinical and clinical groups was 0.90 and 0.89, respectively; test–retest stability was likewise strong (r 5.94) for Turkish version. Brown et al. (2000) showed the validity of the BDI scores for predicting eventual suicide in a 20‐year study of psychiatric outpatients.

#### 2.2.2 Suicide Probability

The Suicide Probability Scale (SPS) was given to quantify the patient's risk of suicide. SPS, developed by Cull and Gill (1988), is a 36‐item self‐report measure of suicide potential designed for use with adolescents and adults. Items are rated on a 4‐point Likert scale (Cull and Gill 1988). Respondents indicate the frequency with which they experienced a specific emotion or behavior by selecting scale anchors ranging from “none or a little of the time” to “most or all of the time.” The SPS consists of four empirically derived subscales for predicting suicidal behavior: (1) hopelessness (12 items), (2) suicide ideation (8 items), (3) negative self‐evaluation (9 items), and (4) hostility (7 items). Validity and reliability studies for the Turkish version were conducted by Atlı, Eskin and Dereboy (2009). For the Turkish version of the SPS, Cronbach's alpha was found to be 0.89.

#### 2.2.3 Hopelessness

The Beck Hopelessness Scale (BHS) was given to evaluate the hopelessness level of the participants. The BHS developed by Beck et al. (1974) aims to assess an individual's level of pessimism about their future (Beck et al. 1974). The scale consists of 20 items that reflect feelings and thoughts about the future. When answering the BHS, individuals are asked to mark “true” for statements that apply to them and “false” for statements that do not. The statements that make up the BHS are divided into three subdimensions: emotions about the future, loss of motivation, and expectations about the future. The total score obtained from the scale reflects the individual's level of hopelessness, which ranges from 0 to 20. The reliability and validity of the BHS have been studied by Seber et al. (1993), and the validity of the scale has been studied by Durak and Palabıyıkoğlu (1994). In this study, the internal consistency coefficient of the scale was 0.92.

#### 2.2.4 Affective Personality Profiles

The affective personality traits of the participants were assessed with ANPS. The ANPS is a questionnaire that measures six basic affects (PLAY, SEEK, CARE, FEAR, ANGER, and SADNESS) and ‘‘Spirituality’’ consisting of 110 items in total (Davis, Panksepp and Normansell 2003). Each subscale includes 14 questions, with 7 positively and 7 negatively formulated, except for the Spirituality subscale, which comprises 12 questions with 6 positively and 6 negatively formulated. The scale includes 14 filler items that assess deception, such as “I always tell the truth.” Respondents answer all questions on a 4‐point Likert scale. The Turkish validity and reliability of the ANPS were conducted by Özkarar‐Gradwohl et al. (2014). Regarding the individual subscales, similar to the original scale, Cronbach's alphas ranged between 0.56 and 0.78.

### 2.3 Statistical Analyses

The data were analyzed using Statistical Product and Service Solutions (SPSS) Statistics 24 (SPSS, Chicago, IL, USA). The normality of the data was assessed using the Kolmogorov–Smirnov (K–S) test, skewness–kurtosis analysis, and graphical methods (see Table S1). The Student's *t*‐test was used for groups where continuous variables were normally distributed, whereas the Mann–Whitney *U* test was used for nonnormally distributed variables. A chi‐square test was carried out to assess differences between categorical variables. Correlation analyses were performed using either Pearson or Spearman tests, depending on the distribution of the data. SPSS macroprocess, which was specifically developed for assessing complex models, including mediators, was used to analyze the data. Subsequently, serial mediation analyses were performed using model six of Hayes’ PROCESS (Version 4.1) macro in order to run the planned analysis of the mediation model (Hayes 2017). Process (Model 6) calculates a test of specific indirect effects through both mediators (in serial) and specific indirect effects through each mediator alone. The models were examined using 5000 bootstrapped samples, with a random sampling method to allow for repeated bootstrapping across all analyses, as recommended by Hayes. In the bootstrap technique, a new observation set is created by repeating the observations in the original data set, and statistical calculations are made with these new data sets. In this method, more reliable results are obtained by correcting the bias and skewness related to the distribution. Confidence interval values are usually reported in bootstrap analysis. Whether there is a mediation effect or not is decided by looking at the values in the 95% confidence interval obtained as a result of the bootstrap analysis. If the confidence interval values do not cover the value of 0, it is understood that the mediation effect occurs (Gürbüz 2019).

## 3 Results

### 3.1 Sociodemographic and Clinical Characteristics of Groups

The study population comprised 160 subjects (age = 31.1 ± 11 years; 105 females) who had been diagnosed with MDD. Among them, 93 (58%) had suicidal ideation (depression with suicide; DS), whereas 67 had no suicidal ideation in their lifetime (depression with no suicidal ideation; DNS).

There were no statistically significant differences in terms of age, gender, marital status, employment status, education level, psychiatric illness history in the family, and suicide history in family between the DS and DNS groups. Only two clinical variables were comparable across patients with and without suicidal ideation. Patients with suicide ideation had more psychiatric medication history (68% vs. 37%) and more suicide attempt history in their family (20% vs. 8%). Table 1 presents the comparison of sociodemographic and clinical characteristics of the study groups.

**TABLE 1:** Socio‐demographical and clinical characteristics of the study groups.

Characteristics	DS (*N* = 93)	DNS (*N* = 67)	*Z* score/chi	*p* value
Age median (IQR)^a^	26 (15)	29 (15)	− 0.615	0.539
Gender female *n* (%)^b^	60 (64.5)	45 (67.2)	0.121	0.728
Marital status^b^, ^d^				
Married/partnered *n* (%)	25 (27.5)	24 (38.7)	2.139	0.144
Single/seperate/widowed *n* (%)	66 (72.5)	38 (61.3)		
Employment status^b^				
Full or part time job *n* (%)	35 (37.6)	28 (42)	0.282	0.595
Nonemployed/retired/student/housewife *n* (%)	58 (62.4)	39 (58)		
Educational level^b^				
Secondary school and lower *n* (%)	34 (36.6)	21 (31.3)	0.324	0.569
College/university *n* (%)	59 (63.4)	46 (68.7)		
Psychiatric medication history^b^, ^d^	56 (67.5)	24 (37)	14.01	< **0.001** *
Psychiatric disorder in the family *n* (%)^b^, ^d^	62 (97)	44 (93.6)	0.669	0.414
Suicide attempt in the family *n* (%)^b^	19 (20.4)	5 (7.5)	5.136	**0.023**
Suicide in the family^c^	5 (5.4)	2 (3)		0.700
Traumatic events median (IQR)^a^	1 (1)	1 (2)		0.446

*Note*: Bold element is used in the meaning of statistically significant.

Abbreviations: DS, depression with suicide idea group; DNS, depression no suicide idea group.

^a^ Mann–Withney *U* test.

^b^ Pearson chi‐square.

^c^ Fischer exact test.

^d^ Missing data were 7 for marital status (DS:2, DNS:5), 12 for psychiatric medication history (DS:10, DNS:2), and 49 for psychiatric disorder family history (DS:29, DNS:20).

### 3.2 Univariate Comparisons of Measures Between Two Diagnose Groups

Univariate comparison by Mann–Whitney *U* test showed that hopelessness scores were higher in the DS group. Moreover, the Student *t*‐test revealed that both depression and suicide probability scores were higher in the DS group. Univariate comparisons revealed that among the ANPS domains, only the mean scores for ANGER were higher in the DS group, whereas there was no statistically significant difference observed in the other sub‐domains. Table 2 presents the univariate comparison of measurement of the study groups.

**TABLE 2:** Univariate comparison of measurement of the study groups.

Measure	Depression with suicide idea	Depression without suicide idea	*Z*/*t*	*p* value
BDI (mean ± SD)^a^	29.1 ± 12	22.4 ± 10.8	− 3.65	**< 0.001**
BHS (median [IQR])^b^	14 (9)	10 (11)	− 2.721	**0.007**
SPS (mean ± SD) ^a^	89 ± 19.8	82.3 ± 20.8	2.07	**0.040**
SEEK (mean ± SD) ^a^	23.2 ± 6	22.2 ± 4.5	− 1.256	0.101
FEAR (mean ± SD)^a^	27.7 ± 7.1	25.5 ± 5.8	− 2.11	0.097
CARE (mean ± SD) ^a^	27.8 ± 6.3	26.6 ± 6.4	− 1.196	0.233
ANGER (mean ± SD) ^a^	28.8 ± 8	25.2 ± 5.7	− 3.110	**0.002**
PLAY (mean ± SD) ^a^	21 ± 5.2	20.7 ± 5.4	− 0.233	0.816
SADNESS (mean ± SD)^a^	25.8 ± 5.7	24 ± 6	− 1.80	0.071
SPIRITUALITY^c^ (median (IQR)^b^	17 (10)	18.5 (7)	− 1.875	0.061

*Note*: Bold element is used in the meaning of statistically significant.

Abbreviations: BHS, Beck Hopeless scale; BDI, Beck depression inventory; SPS, suicide probability scale.

^a^ student *t*‐test.

^b^ Mann–Whitney *U* test.

^c^ One missing data in spirituality (DS:0, DNS:1).

### 3.3 Correlations

As seen in Table 3, Spearman rank correlations between BDI and ANPS scores showed that the BDI score is significantly negatively correlated with SEEK, SPIRITUALITY, and PLAY and significantly positively correlated with FEAR, ANGER, and SADNESS (*p* < 0.001 for all results). BHS scores were significantly negatively correlated with SEEK, PLAY, and SPIRITUALITY and positively correlated with FEAR, ANGER, and SADNESS (*p* < 0.001 for all results). SPS scores were significantly negatively correlated with PLAY and SPIRITUALITY, and there was a positive correlation with FEAR, SADNESS, and ANGER (*p* < 0.001 for all results). No significant correlations were detected among BDI, BHS, SPS scores, and CARE sub‐domain.

**TABLE 3:** Correlation analyses.

	SEEK	FEAR	CARE	ANGER	PLAY	SADNESS	SPIRITUALITY^#^
BDI	***r*** = 0.277**	***r*** = 0.357**	*r* = −0.18	***r*** = 0.248**	***r*** = −0.326**	***r*** = 0.386**	***r*** = −0.223**
BH	***r*** = 0.223**	***r*** = 0.201**	*r* = −0.103	***r*** = 0.128	***r*** = −0.357**	***r*** = 0.265**	***r*** = −0.312**
SPS	*r* = −0.81	***r*** = 0.253**	*r* = 0.007	***r*** = 0.239**	***r*** = −0.130	***r*** = 0.273**	***r*** = −0.271**

*Note*: Bold element is used in the meaning of statistically significant.

Abbreviations: BHS, Beck Hopeless scale; BDI, Beck depression inventory; SPS, suicide probability scale.

**p < 0.001.

^#^ One missing data in spirituality (DS:0, DNS:1).

### 3.4 Mediation Analysis

**FIGURE 1:**
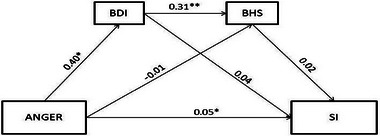
Serial mediation analyses of BDI and BHS between Anger and SI. The results are based on PROCESS Model 6. Beta is not a standardized coefficient. BHS, Beck hopeless scale; BDI: Beck depression inventory; SI: suicidal ideation. *p < 0.05, **p < 0.001.

In the series mediation model, suicidal ideation is an outcome variable, ANGER is a predictive variable, and BDI and BHS are mediators. In serial mediation models, there are four possible pathways linking ANGER to suicide. The first indirect pathway is through BDI (M1). The second indirect pathway is through BHS (M2). The third indirect pathway is through BDI (M1), followed by BHS (M2), in serial. The final pathway is the direct pathway from ANGER to suicide risk. The results of the series mediation model are presented in Table 4 and Figure 1. As shown in Table 4 and Figure 1, ANGER was significantly and positively related to BDI (*b* = 0.40, *p *= 0.0015); BDI was significantly and positively related to BHS (*b* = 0.31, *p* < 0.001). The bootstrapping analyses show that the indirect effect of ANGER on suicidal ideation via BDI was 0.015, and the 95% confidence interval did not contain zero (95%CI = [0.0001, 0.037]).

**TABLE 4:** Serial mediation effect thought Beck depression inventory (BDI) and Beck Hopeless scale (BHS) on the relationship between ANGER and suicidal ideation (SI).

Model pathways	Coefficient *b*	SE	*T*	*p*	LL 95%CI	UL 95%CI
ANGER → BDI	0.40	0.12	3.22	0.0015*	0.15	0.64
ANGER → BHS	− 0.01	0.05	− 0.23	0.816	− 0.11	0.87
BDI → BHS	0.31	0.03	9.97	< 0.001**	0.25	0.37
BDI → SI	0.04	0.02	1.92	0.054	− 0.007	0.07
BHS → SI	0.02	0.04	0.65	0.654	− 0.05	0.10
Direct effect	0.05	0.02	2.14	0.03*	0.004	0.10
Total indirect effect	0.017	0.01			0.003	0.038
ANGER → BDI → SI	0.014	0.01			0.0001	0.037
ANGER → BHS → SI	− 0.0003	0.002			− 0.005	0.004
ANGER → BDI → BHS → SI	0.003	0.005			− 0.006	0.014

*Note*: Insignificant pathways are noted in italics and bold (95% confidence interval contains zero). All pathways are unstandardized. Indirect effects were computed using 5000 bootstrap samples.

Abbreviation: SI: suicidal ideation.

*p < 0.05.

**p < 0.001.

## 4 Discussion

In this study, we investigated the primary affective systems associated with suicidal ideation in individuals with major depression and found the ANGER primary affective system predicts the probability of suicide ideation in depressive patients, and the severity of depression also strengthens the relationship between ANGER and suicide ideation.

Although coping with internal and external stresses, our affective system has a great influence on our internal regulation, behavior, and decisions. Primary affective systems originate from subcortical tissues and shape the cortex with the external world and internal stimuli over time (Panksepp 2011). According to the basic emotion approach, the loss of a loved one or thing will increase the activation of the SADNESS system. The sustained activation of SADNESS increases hopelessness and leads to a transformation from SADNESS to depressive disorders. In this context, the acute period of stimulation of the SADNESS system involves the activation of SEEK. If the stimulation of SADNESS persists, SEEK activities may decrease and progress to a phase of hopelessness characterized by an emotional shutdown (Panksepp and Watt 2011). In our study, hopelessness was associated with low levels of SEEK and PLAY and high levels of FEAR, ANGER, and SADNESS. These results are consistent with Panksepp's theory.

In our study, depressive symptoms were negatively correlated with SEEK and PLAY and positively correlated with FEAR, ANGER, and SADNESS. Panksepp and Watt attributed depression to an imbalance among the SEEKING, PLAY, and SADNESS systems and claimed that the activity of SEEKING and PLAY would decrease and the activity of SADNESS would increase when the depressive tendency was increased. In subsequent studies, it has been observed that SADNESS, FEAR, and ANGER play a fundamental role in depressive tendencies and there is an increase in activation in these regions and a decrease in activation in SEEKING and PLAY (Fuchshuber et al. 2019; Montag and Panksepp 2017). In our study, changes were observed in similar areas as depressive symptoms increased. Patients with and without suicidal ideation differed in the ANGER primary affective system.

The ANGER system defends life resources, such as your own or the lives of those you love, which can also be activated by physical restriction (Montag, Elhai, and Davis 2021). The ANGER subscale is characterized by irritation, frustration, and verbal and physical displays of anger (Brienza et al. 2022). Suicidal ideation has long been thought to be significantly influenced by anger, agression, and hostility, or an emotional state consistent with these emotions (Cui et al. 2023; Goldney et al. 1997; Wilks et al. 2019). Several studies found a strong association between depression and anger in both clinical and normal populations (Balsamo 2010; Koh, Kim, and Park 2002). Suicidal ideation is correlated with anger and anger expression (Park et al. 2010); adolescents in hospital settings who have attempted suicide in the past have higher levels of trait anger than adolescents who are not suicidal (Kelley et al. 1996). In a study conducted in outpatients with major depression, anger attacks were associated with chronic suicide ideation (Jha et al. 2021). In a large participant study, that investigated the predictors of suicidal ideation in terms of sociodemographic characteristics, depression, self‐esteem, and anger. Jang et al. (2014) found that anger was significantly positively correlated with suicide ideation in all age groups, independent of the effects of depression and self‐esteem, and they highlighted interventions for anger will also have an important place in reducing suicidal behavior. The results of our study also support the relationship between anger and suicide ideation.

Similarly, in studies conducted with the affective neuroscience scale, high scores of ANGER have been found in Bipolar‐II disorder and depression (Savitz, van der Merwe, and Ramesar 2008). High ANGER scores are closely associated with anxious depression (Brienza et al. 2022). Considering that the presence of anxiety in patients with depression is a condition that increases suicidal ideation, it supports the view that increased activation of the ANGER system may be associated with increased suicidal ideation in depression (Seo et al. 2011; Wolfersdorf 1995). Consistent with this, according to a recent study, anxiety symptoms in depressed patients modulate the relationship between hopelessness and suicide ideation (Baryshnikov et al. 2020).

Personality trait has been seen as an important predictor for suicide research (Baertschi et al. 2018; Donnellan, Hill, and Roberts 2015; Ferguson 2010). In studies with Five Factor Theory, which is a well‐established and validated theory of general personality, consistently neuroticism has been found to be associated with suicidal ideation (Chioqueta and Stiles 2005; DeShong et al. 2015; Velting 1999). According to Chioqueta and Stiles (2005), high neuroticism, openness to experience, and low extraversion can predict suicide ideation. DeShong et al. (2015) revealed that neuroticism was associated with both current ideation and a history of suicide ideation. In accordance with the results of their study, individuals with high levels of neuroticism and low levels of extraversion may be more prone to suicide ideation. They interpreted this finding as individuals with high levels of neuroticism may be more likely to experience suicide ideation because they are more open to experiencing negative emotions. In addition, individuals with high extraversion may receive more social support due to their tendency to participate in activities that may involve other individuals. Chioqueta and Stiles (2005) examined the relationship between personality and hopelessness and observed that low extraversion and high neuroticism were predictive of hopelessness. Similarly, Duberstein et al. (2001) reported that in a sample of depressed older adults, neuroticism was significant predictor of hopelessness scores, and in addition, there was a negative relationship between hopelessness and extraversion. In the meta‐analysis examining the relationship between the Five Factor Model and ANPS, higher FEAR, ANGER, and SADNESS have been linked to neuroticism, whereas SEEKING was related with openness to experience and PLAY was related with extraversion (Marengo et al. 2021). The fact that ANGER was high and correlated with suicidal ideation in our study is consistent with these findings. Because both groups in our study have depressive disorder, there may not have been a significant difference between the two groups in terms of PLAY, SEEK, FEAR, and SADNESS.

Suicide is often considered an attempt to escape unbearable feelings and mental pain. Studies have revealed that persons with suicidal ideas experience more mental pain (Pompili 2019; Pompili, Erbuto et al. 2022; Pompili, Innamorati et al. 2022). Mental pain can also increase the perceived burdensomeness (LeRoy et al. 2018). Anger is linked to suicidal ideation through perceived burdensomeness and increased acquired capability for suicide through painful and provocative events (Hawkins et al. 2014). Anxiety, agitation, and irritability are also frequently associated with a suicidal crisis (Jobes 2000). This literature also supports the relationship between suicidal ideation and ANGER.

Although it has strengths, this study also has limitations. First, the study does not provide direct evidence for the causal mechanisms underlying the relationship between primary affective systems and suicide ideation. However, it does lay the groundwork for the generation of new hypotheses. To clarify the relationship between affective personality traits and suicide, future follow‐up studies may provide more conclusive results. Moreover, studying affective personality traits may offer insights into the neural mechanisms underlying depression and suicidal ideation. Although no neurostimulation or neuroimaging was performed in our study, it may provide inspiration for consistent new studies on the basis of the studies on ANPS and suicide in the literature. Second, the use of self‐report scales may introduce bias in the prediction of depression severity and the assessment of suicide risk. However, an important strength of this study is that patients were diagnosed with depression by a clinician's examination, not by a scale. A notable limitation is the lack of investigation on impulsivity and suicide attempts. Impulsivity may mediate between affective personality traits such as ANGER and SEEK, and suicide attempts. This relationship seems to be unexplored in the current literature. Therefore, separating suicidal ideation from suicide attempts may provide valuable insights for future research. Finally, the effect of episode number on suicidal ideation and primary emotion system was not examined. Examining the number of episodes in further studies will facilitate the understanding of this relationship.

## 5 Conclusion

In sum, we aimed to investigate the associations between affective systems assessed with ANPS to individual differences in suicidal behavior in depressive patients. In‐line with the affective neuroscience approach, higher trait ANGER scores are associated with suicide ideation. It takes a long time for the primary affective system to shape the cortex, allowing for early detection of underlying risky mechanisms and intervention. These results may be useful for both neuroscientists and clinicians to present a roadmap in which emotional brain networks and associated neurochemical systems may deserve attention. This trait profile can also be used to identify clinically risky patients.

## Author Contributions

**Yasemin Hoşgören Alici**: conceptualization, methodology, data curation, investigation, validation, formal analysis, writing–original draft, writing–review and editing, project administration. **Selvi Ceran**: methodology, data curation, writing–review and editing, writing–original draft, validation, formal analysis, supervision, project administration. **Jamal Hasanli**: methodology, investigation, writing–review and editing, writing–original draft, data curation, formal analysis, validation, visualization, project administration. **Gonca Asut**: data curation, writing–review and editing, project administration. **Beren ÖZEL**: data curation. **Zehra Ucar Hasanli**: data curation. **Gökçe Saygi**: data curation. **Arda Bağcaz**: writing–review and editing, data curation. **Emre Misir**: data curation, writing–review and editing, formal analysis.

## Conflicts of Interest

The authors declare no conflicts of interest.

### Peer Review

The peer review history for this article is available at https://publons.com/publon/10.1002/brb3.70077.

## Supporting information




**Table S1** Kolmogorov–Smirnov normality tests results and Kurtosis and skewness values for the variables.

## Data Availability

The data that support the findings of this study are available from the corresponding author upon reasonable request.
