# Supplementary material for: Why Do Some Depressive Patients Have Suicidal Ideation but Others Not? Suicidal Ideation From the Perspective of Affective Neuroscience Personality Traits
Source: Brain Behav. 2024 Oct 8;14(10):e70077. doi: 10.1002/brb3.70077 (PMC11460610; doi:10.1002/brb3.70077)
Supplement: Supplementary file 1 — Table S1 Kolmogorov–Smirnov normality tests results and Kurtosis and skewness values for the variables. [file BRB3-14-e70077-s001.docx]

**Supplementary table 1**. Kolmogorov-Smirnov normality tets results and Kurtosis and skewness values for the variables

|  | DS | | | | | DNS | | | | |
| --- | --- | --- | --- | --- | --- | --- | --- | --- | --- | --- |
|  | Kolmogorov-Smirnov | Kurtosis | Std. Error | Skewness | Std. Error | Kolmogorov-Smirnov | Kurtosis | Std. Error | Skewness | Std. Error |
| BDI | 0.200* | 0.191 | 0.495 | -0.386 | 0.250 | 0.200* | -0.464 | 0.578 | 0.107 | 0.293 |
| BHS | 0.006 | -0.787 | 0.495 | -0.539 | 0.250 | 0.032 | -1.311 | 0.578 | -0.036 | 0.293 |
| SPS | 0.200* | -0.439 | 0.495 | -0.189 | 0.250 | 0.200* | -0.430 | 0.578 | 0.400 | 0.293 |
| SEEK | 0.010 | 0.600 | 0.495 | -0.272 | 0.250 | 0.200* | -0.204 | 0.578 | 0.223 | 0.293 |
| CARE | 0.200* | -0.361 | 0.495 | -0.015 | 0.250 | 0.200* | -0.690 | 0.578 | 0.029 | 0.293 |
| PLAY | 0.058 | 0.121 | 0.495 | 0.178 | 0.250 | 0.036 | 0.274 | 0.578 | 0.054 | 0.293 |
| FEAR | 0.200* | 1.106 | 0.495 | -0.759 | 0.250 | 0.200* | -0.352 | 0.578 | 0.212 | 0.293 |
| ANGER | 0.200* | -0.620 | 0.495 | -0.136 | 0.250 | 0.042 | -0.545 | 0.578 | 0.242 | 0.293 |
| SADNESS | 0.200* | -0.247 | 0.495 | -0.136 | 0.250 | 0.036 | -0.020 | 0.578 | 0.260 | 0.293 |
| Spirituality | 0.037 | -0.514 | 0.495 | -0.166 | 0.250 | 0.200* | 1.263 | 0.578 | -0.443 | 0.293 |
| Age | 0.000 | 0.502 | 0.495 | 1.101 | 0.250 | 0.003 | -0.260 | 0.578 | 0.849 | 0.293 |

DS: depresssion with suicide idea group DNS: depression without suicide idea group
